# The Impact of Prescribing Monitoring Policy on Drug Use and Expenditures in China: A Multi-center Interrupted Time Series Study

**DOI:** 10.34172/ijhpm.2023.7343

**Published:** 2023-06-20

**Authors:** Xiaoyan Nie, Ruilin Wang, Guangkai Liang, Xinyi Zhang, Ningjia Tang, Yuchun Cai, Congxiao Han, Yuxuan Zhao, Tong Jia, Fang Zhang, Sheng Han, Xiaodong Guan, Luwen Shi, Christine Y. Lu

**Affiliations:** ^1^Department of Pharmacy Administration and Clinical Pharmacy, School of Pharmaceutical Sciences, Peking University, Beijing, China; ^2^Department of Population Medicine, Harvard Medical School and Harvard Pilgrim Health Care Institute, Boston, MA, USA

**Keywords:** Prescribing Monitoring Policy, Over-used drugs, Policy Evaluation, Interrupted Time Series, China

## Abstract

**Background:** A prescribing monitoring policy (PMP) was implemented in November 2015 in Anhui province, China, the first province to pilot this policy to manage the use and costs of select drugs based on their large prescription volumes and/ or costs in hospitals. This study evaluated the impact of PMP on the use and expenditures of different drugs in three tertiary hospitals in Anhui.

**Methods:** We obtained monthly drug use and expenditures data from three tertiary hospitals in Anhui (November 2014 through September 2017). An interrupted time series (ITS) design was used to estimate changes in defined daily doses (DDDs per month) and drug expenditures (dollars per month) of policy-targeted and non-targeted drugs after PMP implementation. Drugs were grouped based on whether they were recommended (recommended drugs) by any clinical guidelines or not (non-recommended drugs), or if they were potentially over-used (proton pump inhibitors, PPIs).

**Results:** After PMP, DDDs and costs of the targeted PPIs (omeprazole) declined while use of non-targeted PPIs increased correspondingly with overall sustained declines in total PPIs. The policy impact on recommended drugs varied based on whether the targeted drugs have appropriate alternatives. The DDDs and costs of recommended drugs that have readily accessible appropriate alternatives (atorvastatin) declined, which offset increases in its alternative non-target drugs (rosuvastatin), while there was no significant change in those recommended drugs that did not have appropriate alternative drugs (clopidogrel and ticagrelor). Finally, the DDDs and costs of non-recommended drugs decreased significantly.

**Conclusion:** PMP policy impact was not the same across different drug groups. PMP did help contain the use and costs of potentially over-used drugs and non-recommended drugs. PMP did not seem to reduce the use of first-line therapeutic drugs recommended by clinical treatment guidelines, especially those lacking alternatives; such drugs are unlikely appropriate candidates for PMP.

## Background

**Key Messages**

**Implications for policy makers**

- Government-led prescribing monitoring policy (PMP) in China can be an effective way to reduce drug use for most utilized and/or highest costs drugs in general.
- PMP can reduce the use of potentially clinically inappropriate and/or overused drugs.
- PMP did not seem to reduce the use of first-line therapeutic drugs recommended by clinical treatment guidelines, especially those lacking alternatives; such drugs are unlikely appropriate candidates for PMP.
- Drug monitoring policy that targeted highly used and/or high-cost drugs should involve multi-disciplinary team to promote rational use, rather than applying a one-fit-all monitoring policy targeting drugs solely by their large volume of use or high costs.

**Implications for the public**

We have identified differential impacts of the same policy on different drugs in this study. Our results showed that applying a prescribing monitoring policy (PMP) that targeted the use of most utilized and/or highest cost drugs in hospitals can help contain the use of potentially clinically inappropriate and/or over-used drugs. However, implementing PMP to contain use of guideline-recommended drugs because of their large volume of use or high costs may not be clinically appropriate and risk increasing management costs for hospitals. To better promote the rational use of drugs, policies should be supplemented by patient centered efforts involving a multi-disciplinary team at local hospitals.

High and ever-increasing pharmaceutical expenditure has been a major concern worldwide. The global pharmaceutical expenditure reached $1.2 trillion in 2018^1^ and $1.25 trillion in 2019^2^. Between 2016 and 2020, 29 608.1 billion Chinese yuan (equivalent to US$ 4626.3 billion) had been spent on health care with an average annual increase of 11.1% in China, and drug expenditures accounted for 39.1% of outpatient expenditures and 26.2% of inpatient expenditures in 2020.^3^ As pharmacotherapy is a recommended element for medical care for most patients, irrational use of medicines leads not only to waste of resources but also to potential health hazards. The World Health Organization (WHO) indicates that up to 50% of medicinal products in the world are prescribed, dispensed, or sold improperly.^4^ Globally, some of the most common medicines that are irrationally prescribed are antibiotics,^5^ proton pump inhibitors (PPIs),^6^ and glucocorticoids.^7^

 One of the issues that China’s health system reform aimed to address was the rapid growth of drug expenditures, which has been a key contributor to health expenditure growth.^8^ Zero-markup drug policy, which eliminated the 15% mark-up allowance on prescribed drugs, has been one of the policies mandated by the government to contain health expenditure growth in China. The Zero-Markup Drug Policy was introduced in primary health care facilities in 2009, and mandated for county-level hospitals in 2012 and for city-level hospitals in 2015. Literature shows that the Zero-Markup Drug Policy and altered fee schedules reduced drug expenditures, but unintentionally increased total health expenditures due to increased provision of diagnostic tests and basic medical services.^9^ Thus, focusing on containing the total drug expenditure without considering rational use of medicines does not appear to be sustainable. Supplementary policies have been implemented in pilot provinces along with the health system reform to help mitigate irrational use of drugs, which started with antibiotics and then most used drugs with high volume and/or high costs but might not be appropriate clinically. Prescribing monitoring policy (PMP) is one of the supplementary policies to help contain drug expenditures by curbing the potential of irrational drug use.

 To monitor and promote the rational use of most used drugs with high volume and/or high costs (‘top-ranked’) drugs, a PMP has been implemented since 2015 in 21 pilot provinces in China, first piloted in Anhui province. The PMP was developed by National Health Commission of China, and was piloted in several provinces. The policy targeted top-ranked 20-30 drugs by total costs with high prescription volumes and/or high expenditure, potentially overused drugs (irrational drug use), and those considered “non-recommended.” Non-recommended drugs were those with no specific indications but were used as ancillary drugs (eg, monosialotetrahexosyl ganglioside, a type of glycosphingolipid containing sialic acid, mainly used for stroke as an ancillary drug but is not recommended by any clinical guidelines). Non-recommended drugs are typically selected by pharmacy administrative and therapeutic committees of hospitals based on irrational use of medications detected by pharmacists and drug expenditures. PMP principle was the same in different provinces but the list of drugs affected by PMP differed across provinces based on their baseline use and costs. PMPs in most pilot provinces focused on monitoring non-recommended drugs whose costs were not commensurate with the achieved health effects and were over-used according to local pharmacy administrative and therapeutic committees. For example, PPIs were targeted by PMP because of their sharply increased use in hospitals and for being overused as prophylaxis treatments in some provinces. Overuse of prophylaxis treatments and non-recommended auxiliary drugs in China rose from a legacy of the past when revenues of healthcare providers were closely related to volumes of drugs prescribed.^10^

 Anhui province was the first province to implement PMP in China to promote the rational use of a long list of drugs in hospitals. In July 2015, the Provincial Health Commission of Anhui province recommended the establishment of the province’s PMP system. The policy stated that all public hospitals in Anhui province shall notify healthcare providers of most used drugs and monitor specific products from different manufacturers for unusual increases in volume and costs compared to historical patterns. Subsequently, in November 2015, a PMP drug list was established according to the purchasing data at the provincial level and the potential for irrational use as suggested by an expert panel from the PATC in Anhui, and statins were added to the list in August 2016. The PMP list included 30 drugs for public secondary hospitals and 20 drugs for primary care institutions. The 30 drugs in PMP list to be monitored in hospitals not only included non-recommended drugs but also guideline-recommended drugs such as statins that were among the most used drugs with high volume and costs. The use of drugs on the PMP list were strictly monitored by pharmacists and PATC in hospitals.

 Since its introduction, the policy has been criticized for its stringent restriction on the use of drugs on the PMP list. Thus, many other provinces that implemented PMPs after Anhui province mostly focused on non-recommended drugs and excluded any drugs that had been recommended by at least one clinical guideline. During the pilot period, the PMP list in Anhui included guideline-recommended drugs and non-recommended drugs among the top-ranked drugs by total costs. Little is known, however, about how PMP influences the use and expenditures of targeted (affected by the policy) and non-targeted (not affected by the policy) drug products, and whether guideline-recommended and non-recommended drugs may be affected differentially by PMP in Anhui. The aim of study was to evaluate the impact of PMP on the use and expenditure of targeted and non-targeted medicines in Anhui.

## Methods

###  Data Source

 Monthly prescription volume (number of packages) and expenditure data between November 2014 and September 2017 were collected from three major tertiary hospitals in Anhui province. The average beds of the three studied hospitals were 3888 ± 1565, and the average number of inpatient and outpatient visits was 298 ± 183 million per year.

###  Study Drugs 

 To evaluate the differential effects of PMP on recommended and non-recommended drugs, PMP targeted drugs were grouped as “recommended drugs” (group R) and “non-recommended drugs” (group N) based on whether they were recommended by any clinical guidelines. PPIs which were monitored as potentially over-used drugs were listed as a particular group (group P). Study drug groups are shown in Table 1: (*i*) PPIs (group P), which were recommended as first-line therapies by at least one international and local clinical guidelines and were monitored by PATC as overused prophylaxis therapy. Omeprazole from one specific manufacturer was targeted by PMP, which accounted for most of PPI prescriptions in Anhui. (*ii*) Recommended drugs (group R) on the PMP list that were recommended as first-line therapies by at least one international and local clinical guidelines. Atorvastatin and clopidogrel were included in the study as these were the only two drugs for chronic diseases on the PMP list that met the qualification as recommended drugs. (*iii*) Non-recommended drugs (group N) on the PMP list whose costs were not commensurate with the achieved health effects and were deemed over-used by PATC in hospitals and were not recommended by any international or local clinical guidelines. Monosialotetrahexosyl ganglioside and oxiracetam were selected for the study as they were heavily prescribed non-recommended drugs.

**Table 1:** Study Drugs in Different Groups

**Targeted Medicine Group**	**Targeted Drugs**	**Non-targeted Drugs**
Group P (PPIs)	Omeprazole T	Omeprazole N Other PPIs: lansoprazole, pantoprazole, rabeprazole, esomeprazole
Group R (Recommended drugs)	Atorvastatin	Rosuvastatin
	Clopidogrel	Ticagrelor
Group N (Non-recommended drugs)	Monosialotetrahexosyl ganglioside, oxiracetam	-

Abbreviation: PPIs, proton pump inhibitors.

 We compared PMP’s effects on the above three drug groups. We also compared changes in the use and expenditures of policy-targeted (listed on the PMP list) and non-targeted (not on the PMP list) products in each group except for group N. We paired non-targeted drugs to each targeted drug on the PMP list according to their similarities in pharmacological effects, clinical indications, and target populations. Use of non-targeted drugs could remain the same following the PMP as they were not exposed to the policy, or their use could change as they were substituted for the PMP-targeted drugs. We did not identify non-targeted drug pairs for group N drugs as drugs categorized into this group were all non-recommended drugs, without clearly established clinical indications or recommendations from international or local clinical guidelines.

 Typically, a drug with generic substitutes available can be supplied by at most two manufacturers in public hospitals in China. In Anhui, two manufacturers supplied omeprazole, and one product was on the PMP list (ie, omeprazole targeted, referred to as “omeprazole T” hereafter) while the other (omeprazole non-targeted, “omeprazole N” hereafter) was not. All the other four PPIs used in Anhui (lansoprazole, pantoprazole, rabeprazole, and esomeprazole) were also not targeted by the policy.

 For group-R drugs, rosuvastatin was paired with atorvastatin as the non-targeted counterpart as they were considered therapeutic alternatives by the PATC and these two statins accounted for more than 90% of the prescribed volume of all statins in Anhui. Ticagrelor was chosen as the non-targeted drug and was paired with clopidogrel as they were the only two oral P2Y12 inhibitors on the market in Anhui.

 For group N drugs, there were no appropriate non-targeted, alternative drug pairs. Group N drugs were those drugs used to treat many diseases despite having no specific clinical indication or recommendation by international or national clinical guidelines. Thus, it is challenging to select appropriate alternative drugs that would be used by similar patients.

###  Outcome measures

 We estimated defined daily doses (DDDs) per month for use^11^ from number of packages (total mg) and monthly expenditures of each study drug. Costs were reported in Chinese RMB and adjusted for inflation using the medical care component of the Price Index.^12^

###  Study Design and Statistical Analysis

 We conducted an interrupted time series (ITS) analysis,^8^ a strong quasi-experimental study design that is robust against most threats to internal validity^9^ through controlling for secular trends in study outcomes. The method assessed whether PMP caused abrupt changes in the level and/or the pre-existing trend (slope) of study outcomes.

 Time series of DDDs and costs were divided into two segments: the “pre-PMP” period (from November 2014 to October 2015 for all study drugs except for statins; from November 2014 to July 2016 for statins) and the “post-PMP” period (from December 2015 to September 2017 for all study drugs except for statins; from September 2016 to September 2017 for statins); the policy implementation month (November 2015 for all study drugs except for statins; August 2016 for statins) was removed from the statistical model. We used segmented regression models to estimate level and trend changes in our outcome measures from the pre-PMP period to the post-PMP period.

 The formula was as follows:

*Y*_t_* = β*_0_* + β*_1_* × time*_t_* + β*_2_* × intervention*_t_* + β*_3_* × time after intervention*_t_* + e*_t_

 In this model, *Y*_t_ is the DDDs or costs of study drugs in month t; *time*_t_ is a continuous variable indicating time t in months from the start of the observation period; *intervention*_t_ is an indicator for time *t* occurring before or after the policy; and *time after intervention*_t_ is a continuous variable counting the number of months after the intervention at time t, with 0 denoting the first month following PMP implementation.^10^ *β*_0_ estimatesthe baseline level of outcome measures at time zero; *β*_1_ estimates the change in outcome measures before the intervention, which reflects the baseline trend; *β*_2_ estimates the level change in outcome measures immediately after the intervention; and *β*_3_ estimates the trend change in outcome measures after the policy (“post-PMP”), compared with the monthly trend before the policy (“pre-PMP”). The sum of *β*_1_ and *β*_3_ is the post-intervention slope. *e*_t_ represents the random variability not explained by the model. This model adjusts for baseline level and trend of the outcomes before PMP, allowing us to infer that the observed changes are likely attributable to the policy. We used the Durbin-Watson test to detect autocorrelations^11^ and the autoregression procedure to correct for first order serial correlation when needed.^12^

## Results

###  The Impact of PMP on Use and Costs of Targeted and Non-targeted PPIs 

**Figure 1:**
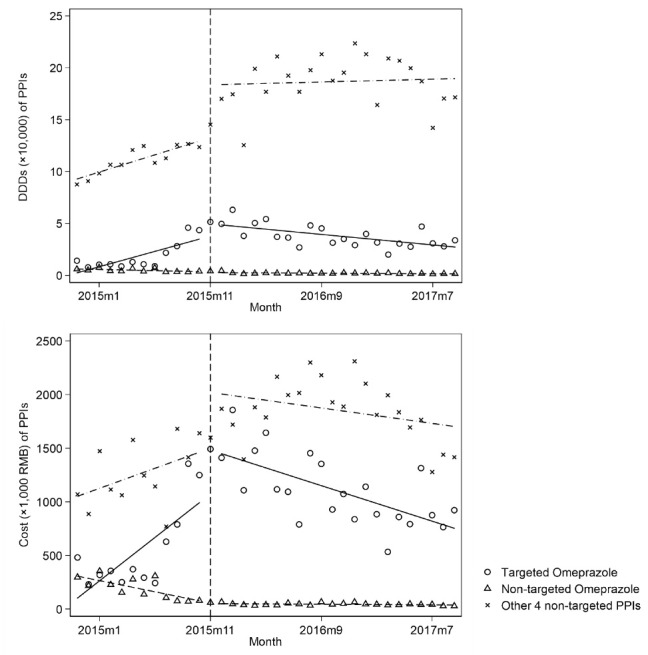
Use in Defined Daily Doses and Costs of Proton Pump Inhibitors Per Month Before and After the Prescribing Monitoring Policy, Initiated in November 2015.

 PPIs were the only drug category on the PMP list that were recommended as first-line therapies by at least one international and local clinical guidelines while at the same time were monitored by PATC as overused prophylaxis therapy. Figure 1 shows the ITS analysis results on the changes in DDDs and costs of targeted and non-targeted PPIs after PMP implementation, and Table 2 shows the statistical results of changes in the use and expenditures of these drugs.

**Table 2:** Trends in the DDDs and Costs of Studied Drugs Before and After the Implementation of Prescribing Monitoring Policy in Anhui

**Drug**		**Baseline**	**95% CI**		**Level Change**	**95% CI**		** Trend Change**	**95% CI**		**Durbin Watson Test**
Omeprazole targeted	DDDs	**2931.0 **	1332.1	4529.9	11664.8	-2309.2	25638.7	**-3939.3 **	-5666.6	-2212.0	1.9
Omeprazole non-targeted	DDDs	**-224.1 **	-354.8	-93.5	-616.0	-1768.5	536.4	**159.3 **	21.3	297.3	2.0
Total omeprazole	DDDs	**2699.0 **	1119.7	4278.2	11231.5	-2584.2	25047.1	**-3777.5 **	-5481.4	-2073.5	1.9
Other 4 non-targeted PPIs	DDDs	**3323.1 **	**2487.3 **	**4158.8 **	**44632.9 **	**20919.6 **	**68346.2 **	**-2632.9 **	**-4613.0 **	**-652.8 **	1.6
Total 5 PPIs	DDDs	**6017.3 **	2338.7	9695.8	**56578.0 **	24166.8	88989.2	**-6433.1 **	-10354.3	-2511.9	2.0
Omeprazole-targeted	Cost	**80360.0 **	33522.6	127197.4	402459.4	-7949.6	812868.3	**-113073.4 **	-163496.1	-62650.8	1.9
Omeprazole non-targeted	Cost	**-20899.4 **	-25723.2	-16075.7	-5213.3	-47663.7	37237.0	**20309.7 **	15236.5	25382.9	2.1
Total omeprazole	Cost	**59834.0 **	15598.2	104069.8	**403221.3 **	14443.3	791999.4	**-93519.9 **	-140918.0	-46121.8	1.9
Other 4 non-targeted PPIs	Cost	37341.9	-1548.7	76232.5	**451524.0 **	**34166.7 **	**868881.3 **	**-47190.3 **	**-90718.4 **	**-3662.2 **	1.7
Total 5 PPIs	Cost	**97467.8 **	31651.0	163284.5	**856478.4 **	277213.1	1435744.0	**-141169.3 **	-211502.6	-70836.0	2.0
Atorvastatin	DDDs	**4347.6 **	2689.8	6005.5	18501.6	-13324.2	50327.5	**-8863.1 **	-12332.1	-5394.2	2.1
Rosuvastatin	DDDs	-201.5	-966.0	563.1	6270.5	-8188.2	20729.1	**4008.2 **	2407.7	5608.7	2.0
Atorvastatin	Cost	**28387.7 **	14151.4	42623.9	223674.0	-49525.3	496873.3	**-78951.1 **	-108738.5	-49163.7	2.1
Rosuvastatin	Cost	1253.4	-6715.3	9222.1	103888.3	-46783.4	254559.9	**26132.3 **	9450.0	42814.6	2.0
Clopidogrel	DDDs	3437.5	-2023.6	8898.6	16039.2	-32086.1	64164.5	-1962.0	-7715.6	3791.6	2.2
Ticagrelor	DDDs	**107.0 **	**54.8 **	**159.2 **	**2683.4 **	**732.1 **	**4634.6 **	15.5	-170.9	201.9	1.4
Clopidogrel	Cost	33946.3	-42711.5	110604.1	169119.8	-507039.7	845279.3	-24449.5	-105391.9	56492.8	2.1
Ticagrelor	Cost	**1280.1 **	**542.2 **	**2018.0 **	**48965.8 **	**2961.5 **	**94970.1 **	1644.6	-1928.5	5217.6	1.4
Oxiracetam	DDDs	**359.6 **	**94.6 **	**624.5 **	**7382.3 **	**3766.9 **	**10997.6 **	**-604.6 **	**-1049.6 **	**-159.7 **	1.6
Monosialotetrahexosyl ganglioside	DDDs	**187.6 **	**34.2 **	**341.0 **	**5686.5 **	**3202.0 **	**8170.9 **	**-545.1 **	**-734.8 **	**-355.5 **	1.4
Oxiracetam	Cost	**47034.1 **	**11458.2 **	**82610.0 **	**297947.7 **	**56758.2 **	**539137.2 **	**-72094.0 **	**-112497.1 **	**-31690.9 **	1.8
Monosialotetrahexosyl ganglioside	Cost	**60061.7 **	**9477.6 **	**110645.8 **	**405188.1 **	**51289.6 **	**759086.7 **	**-93713.0 **	**-148097.7 **	**-39328.4 **	1.8

Abbreviations: CI, confidence interval; PPI, proton pump inhibitors; DDDs, defined daily doses. Data with *P* < .05 were bold. *Note:* Other four non-targeted PPIs referred to lansoprazole, pantoprazole, rabeprazole, and esomeprazole.

 For targeted omeprazole, the use and costs increased rapidly before PMP. There were significant trend changes of -3939 DDDs per month (95% confidence interval [CI]: -5667, -2212) and -113 073 RMB per month [CI: -163 496, -62 651] after PMP implementation.

 In contrast, the use and costs of non-targeted omeprazole declined before PMP. There were significant trend changes of 159 DDDs per month [CI: 21, 297] and 20 309 RMB per month [CI: 15 237, 25 383] after PMP.

 For total omeprazole (targeted and non-targeted), the use and costs increased rapidly before PMP. There was a significant trend change of -3777 DDDs per month [CI: -5481, -2074] after the policy. There was a significant level change of 403 221 RMB [CI: 14 443, 791 999] and a significant trend change of -93 520 RMB per month [CI: -140 918, -46 121] after PMP implementation.

 For the non-targeted PPIs, there was a significant level change of 41 071 DDDs [CI: 5031, 77 111] and a significant trend change of -263 DDDs per month [CI: -4613, -653] after PMP. There was also a significant trend change of -47 190 RMB per month [CI: -90 718, -3662] after PMP implementation.

 For all PPIs, the use and costs increased rapidly before PMP. There was a significant level change of 56 578 DDDs [CI: 24 166, 88 989], and a sustained trend change of -6433 DDDs per month [CI: -10 354, -2512] after PMP implementation. Similar to total omeprazole, there were a significant level change of 856 478 RMB [CI: 277 213, 1 435 744] and a sustained significant trend change of -141 169 RMB per month [CI: -211 502, -70 836] in the total costs of all PPIs after PMP implementation.

###  The Impact of PMP on the Use and Costs of Guideline-Recommended Drugs 

**Figure 2:**
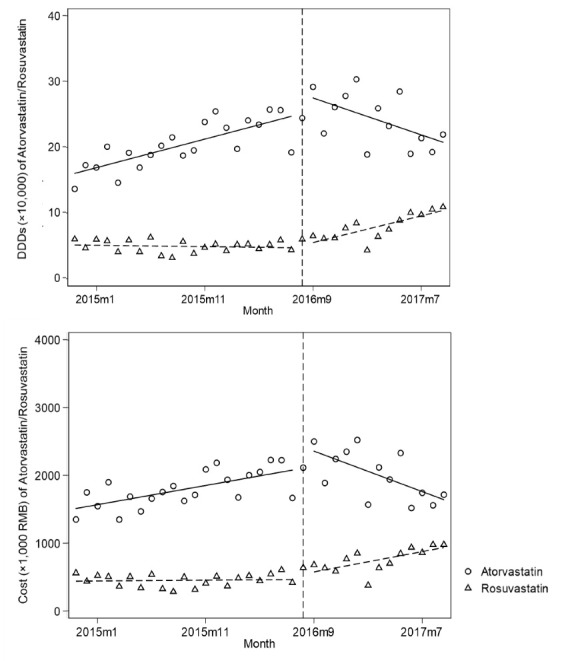
Use in Defined Daily Doses and Costs Per Month of Atorvastatin and its Alternative Drug Rosuvastatin Before and After the Prescribing Monitoring Policy, Initiated in August 2016.

 The ITS analysis results on the changes in DDDs and costs of atorvastatin and its therapeutic alternative, rosuvastatin, before and after the implementation of PMP are shown in Figure 2, and statistical results are shown in Table 2.

 For atorvastatin (a targeted and recommended drug), the use and costs increased rapidly before PMP. After PMP, there was a significant trend change of -8863 DDDs per month [CI: -12 332, -5394] and -78 951 RMB per month [CI: -108 739 -49 164] after PMP implementation.

 For rosuvastatin (non-targeted, a therapeutic alternative to atorvastatin), there was a significant trend change of 4008 DDDs per month [CI: 2408, 5609] after PMP implementation. Correspondingly, there was a significant trend change of 26 132 RMB per month [CI: 9450, 42 814] after PMP implementation.

 For the total use and costs of atorvastatin and rosuvastatin, the level and trend changes were similar with that of atorvastatin.

**Figure 3:**
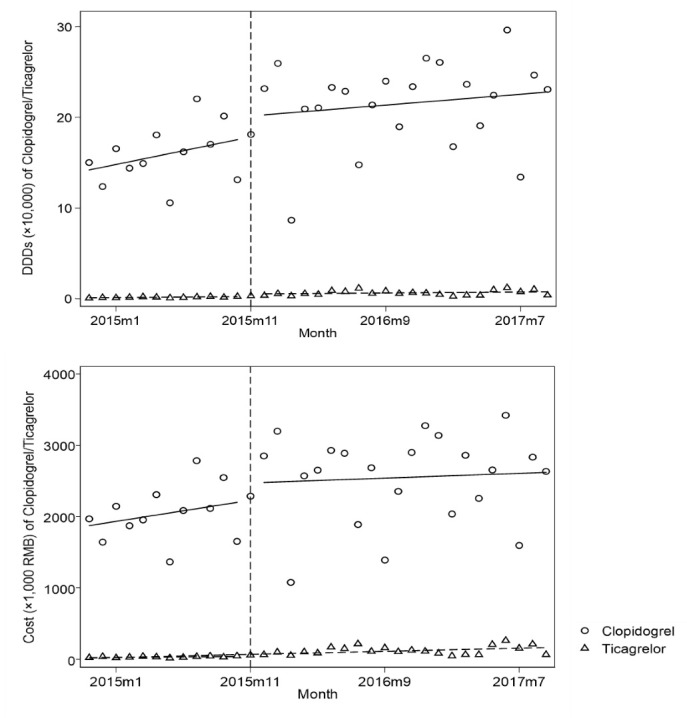
Use in Defined Daily Doses and Costs Per Month of Clopidogrel and its Alternative Drug Ticagrelor Before and After the Prescribing Monitoring Policy, Initiated In November 2015.

 Results of the changes in DDDs and costs of clopidogrel and its therapeutic alternative, ticagrelor, before and after the implementation of PMP are shown in Figure 3, and the statistical results of the ITS analyses are shown in Table 2.

 For clopidogrel (a policy-targeted and recommended drug), the use and costs increased rapidly before PMP. There were no significant level or trend changes in DDDs per month, or in cost after PMP.

 For ticagrelor (non-targeted, a therapeutic alternative to clopidogrel), the use and costs increased rapidly before PMP. There was a significant level change of 2683 DDDs [CI: 732, 4635] but no significant trend changes after PMP implementation. Correspondingly, there was a significant level change of 48 966 RMB in related costs [CI: 2962, 94 970], but the trend change was not significant after PMP implementation. For clopidogrel and ticagrelor altogether, there was no significant trend change in DDDs or in costs.

###  The Impact of PMP on the Use and Costs of Non-recommended Drugs

 Results of the ITS analysis of the DDDs and costs of monosialotetrahexosyl ganglioside and oxiracetam before and after the implementation of PMP are shown in Table 2. In this drug group we did not identify non-targeted drug pairs as they were all non-recommended drugs, without established clinical indications or recommendations from international or local clinical guidelines.

 For targeted, non-recommended drugs, the use and costs increased rapidly before PMP. There were significant level changes of 7382 [CI: 3767, 10 998] and 5687 [CI: 3202, 8171] DDDs and significant trend changes of -605 [CI: -1050, -1606] and -545 [CI: -735, -356] DDDs per month for oxiracetam and monosialotetrahexosyl ganglioside, respectively, after PMP implementation. For oxiracetam, there was a significant level change of 269 020 RMB (CI: 51 886, 486 153) and a sustained trend change of -62 749 RMB per month (CI: -99 855, -25 644) after PMP implementation. For monosialotetrahexosyl ganglioside, there were a significant level change of 365 631 RMB (CI: 51 978, 679 284) and a sustained trend change of -813 829 RMB per month (CI: -121 268, -31 495) after PMP implementation.

## Discussion

 This study investigated the impact of PMP on the use and expenditures of select groups of drugs in three tertiary hospitals in Anhui province. Importantly, we found that PMP had differential impacts on different categories of drugs, suggesting that one PMP does not fit all drug products.

 We found that PMP reduced the use and expenditure of PPIs (potentially overused drugs) significantly through the monitoring of just one single PPI (omeprazole T). PPIs has been widely regarded as safe and well tolerated and were widely used for the treatment and prophylaxis of upper gastrointestinal tract disorders. Given their proven effectiveness and with widespread advertising, the number of dispensed PPIs increased progressively in almost all industrialized countries.^13,14^ The use and expenditure of PPIs increased rapidly in China^10,20^; PPIs are prescribed for indications other than those recommended by expert consensus statements in more than half the cases.^21^ PPI expenditure increased from 1.85 million RMB (US$ 0.29 million) to 7.96 million RMB (US$ 1.23 million) in outpatient settings and from 3.15 million RMB (US$ 0.49 million) to 25.29 million RMB (US$ 3.91 million) in inpatient settings from 2007 to 2016, according to data from a tertiary hospital in China. Studies involving the general population from the United States and the United Kingdom have reported that up to 70% of PPI prescriptions were prescribed without an indication.^22^ Inappropriate uses of PPIs in inpatient and outpatient settings were estimated to cost $12 272 and $59 272 per year, according to data from a tertiary-care teaching hospital in New York, US.^18^ The use of and expenditure on PPIs grew partly due to the irrational use of PPI that could be intervened by pharmacists. The continuous expansion of the PPI market has raised concerns about the possible inappropriate prescribing of these drugs among regulatory authorities of many countries.^15,16,11,23^ PPIs, especially omeprazole, were among the top 20 most frequently used drugs in the three studied hospitals. We found that PMP reduced the use and expenditure of PPIs significantly, though there were small increases in the use of non-targeted omeprazole. After PMP implementation, both the use and expenditures of the targeted omeprazole (omeprazole T) and four non-targeted PPIs (lansoprazole, pantoprazole, rabeprazole, and esomeprazole) as well as the total use and costs of all five PPIs decreased significantly. This indicates that PMP helped contain the total use of PPIs by monitoring just one single PPI (omeprazole T).

 In contrast to PPIs, PMP had less impact on the use and expenditure of guideline-recommended drugs. The use and costs of the targeted drug, atorvastatin reduced significantly after PMP. There were corresponding significant increases in the use of its non-targeted counterpart, rosuvastatin, which was not the intent of the policy. Rosuvastatin was the clinical alternative to atorvastatin, whose availability was similar to atorvastatin in the Chinese market as they were on the same medical insurance reimbursement catalogue. Thus, when PMP required monitoring the use and prescribing of atorvastatin, the use of rosuvastatin increased as a substitute to atorvastatin. This suggests that PMP for atorvastatin did not work as intended (reduce use) because there was significant substitution effect and thus the overall total use and costs for related drugs did not reduce as intended (but clinically appropriate).

 It is worth mentioning that, the impact of PMP on a guideline-recommended drug in another group, clopidogrel (targeted) and its alternative drug ticagrelor (non-targeted), were quite different from those on atorvastatin, and its alternative drug, rosuvastatin. clopidogrel, a guideline-recommended and targeted drug, or its alternative and non-targeted drug, ticagrelor. The use and costs of clopidogrel were resistant to PMP, suggesting that its use was overall clinically necessary and thus the policy had a limited role to play. Clopidogrel and ticagrelor were used in clinical practices with clear indications and were recommended as first-line drugs for patients with coronary artery disease by both international and national clinical guidelines.^17,18^ There are only two oral P2Y12 platelet receptor inhibitors in Chinese market, clopidogrel and ticagrelor. Ticagrelor entered Chinese market in 2012, much later than clopidogrel, with limited health insurance coverage. This might also explain the lack of change in the use and expenditure of ticagrelor. The results shown that PMP might be ineffective in curbing the use of first-line therapeutic drugs recommended by clinical treatment guidelines, especially those lacking alternatives.

 As a contrast to PPIs and group-R drugs, the use and costs of non-recommended drugs (group N drugs), oxiracetam and monosialotetrahexosyl ganglioside, decreased significantly after PMP. We did not examine any alternative drugs for group N drugs as these drugs were used to treat many diseases without specific clinical indications or recommendation by any international or national clinical guidelines. Many non-recommended drugs lack evidence on clinical benefits, were used as auxiliary treatment, and were produced by local manufacturers. Those non-recommended drugs were the main target by PMPs in most provinces.

 By comparing the impact of PMP on PPIs, guideline-recommended drugs and non-recommended drugs, we identified that PMP in Anhui province had differential impacts on different drugs. PMP reduced the use of PPIs, including targeted and non-targeted PPIs, suggesting that the policy helped contain expenditures of those potentially over-used drugs. PMP reduced use and costs of targeted, guideline-recommended drugs such as atorvastatin. However, as they are clinically necessary, there were appropriate and corresponding increases in use and costs of its alternative, non-targeted drugs. When the non-targeted, alternative drugs were not covered by the health insurance (such as the case for ticagrelor), however, PMP did not affect the use of its targeted, recommended counterpart (clopidogrel), indicating that the use of recommended drugs matched clinical needs appropriately. For non-recommended drugs that lacked clinical evidence but were used commonly in clinical practices, PMP reduced their use but there might have been substitution effects pending further investigations. Thus, one policy might not fit all the drugs.

 Our results suggest that reductions in use and costs of different types of drugs by one single policy are not possible and are also not necessarily clinically appropriate. The main purpose of PMP was to promote the appropriate use of drugs by reducing the use of clinically unnecessary drugs, and not merely to reduce the use of policy-targeted drugs. While PMP can help contain use of clinically inappropriate, over-used drugs, we also need a multi-disciplinary team to promote rational use of drugs. This requires better communications among physicians, pharmacists, and nurses to enhance rational drug use and adhere to clinical recommendations. Clinical pharmacists play an important role in promoting the rational use of drugs^26-28^ and have already contributed to the rational use of antibiotics in China.^29-32^ Continued engaging of clinical pharmacists in the implementation of PMP and other strategies is needed to promote the rational use of potentially overused drugs in China.

## Conclusions

 Our analyses of use and drug expenditure of select drugs in three tertiary hospitals in Anhui province suggests that the PMP can help contain the use of potentially clinically inappropriate and over-used drugs. However, deciding and monitoring the use of guideline-recommended drugs because of their large volume of use or high costs may not be clinically appropriate and risk increasing management costs for hospitals. Policies that are supplemented by patient-centered efforts from a multi-disciplinary team at local hospitals might be needed to better promote the rational use of drugs.

###  Limitation

 This study has several limitations. First, our study analyzed data from November 2014 to September 2017, which might not be sufficient to observe the long-term impact of the policy. Our observation ended in September 2017 due to lack of long-term data availability. Also, as the PMP list evolved overtime with the evolving ranking of drug use and costs, some drugs included in this study were excluded from the PMP list after September 2017. Second, we did not identify appropriate alternative, non-targeted drugs for drugs in the non-recommended group because they lacked specific clinical indications. However, this did not influence our study purpose and results as we have included alternative, non-policy targeted drugs for other drug groups (PPIs and recommended drugs: clopidogrel and atorvastatin) for comparison. Third, we did not include other statins to evaluate the impact of PMP on the total use of statins, but the two statins included in the analysis accounted for over 90% of the volume of all statins prescribed in the Anhui province over the study period. Fourth, we did not include histamine type 2 receptor antagonists in our analysis of PPIs. While histamine type 2 receptor antagonists and PPIs are the two main classes of drugs used to treat acid-related disorders, reductions in PPIs might not necessarily affect histamine type 2 receptor antagonists use. Since their introduction in clinical practice, PPI use continues to grow and PPIs have largely replaced histamine type 2 receptor antagonists.^20^ Lastly, we did not have similar data from other Chinese provinces as a comparison. Future studies are needed to investigate changes in medical care elsewhere following changes in drug utilization.

## Acknowledgements

 Luyan Fan, Ling Jiang, Aizong Shen, Weiping Wang from the three studied hospitals were the experts in the Pharmacy Administrative and Pharmacotherapy Committee and helped organizing the data collection of this study. The authors would like to extend thanks to the expert panel who contributed to this study.

## Ethical issues

 The study was considered not human subjects research by Peking University Institutional Review Board.

## Competing interests

 Authors declare that they have no competing interests.
